# Effect of Universal Adhesives on Long-term Bond Strength to Zirconia

**DOI:** 10.3290/j.jad.b3512333

**Published:** 2022-10-24

**Authors:** Renata Vasconcelos Monteiro, Daniela Micheline dos Santos, Bruna Chrispim, Jussara Karina Bernardon, Thiago Soares Porto, Grace Mendonça De Souza

**Affiliations:** a PhD Candidate, Department of Operative Dentistry, Federal University of Santa Catarina (UFSC), Florianopolis, Brazil. Study conception and design, material preparation, data collection and analysis, wrote first draft of the manuscript, read and approved the final manuscript.; b Associate Professor, Department of Dental Materials and Prosthodontics, Sao Paulo State University (UNESP), Sao Paulo, Brazil. Study conception and design, material preparation, data collection and analysis, wrote first draft of the manuscript, read and approved the final manuscript.; c PhD Candidate, Department of Operative Dentistry, Federal University of Santa Catarina (UFSC), Florianopolis, Brazil. Study conception and design, material preparation, data collection and analysis, read and approved the final manuscript.; d Associate Professor, Department of Operative Dentistry, Federal University of Santa Catarina (UFSC), Florianopolis, Brazil. Study conception and design, material preparation, data collection and analysis, read and approved the final manuscript.; e Assistant Professor, Department of Operative Dentistry, College of Dentistry, University of Iowa, Iowa City, IA, USA. Study conception and design, material preparation, data collection and analysis, proofread final version, read and approved the final manuscript.; f Associate Professor, Department of Comprehensive Dentistry, University of Louisville, Louisville, KY, USA. Study conception and design, proofread final version, read and approved the final manuscript.

**Keywords:** dental ceramic, universal adhesive, composite cement, microshear bond strength.

## Abstract

**Purpose:**

To evaluate the effect of universal adhesives on the long-term bond strength to yttria-stabilized tetragonal zirconia polycrystal (Y-TZP).

**Materials and Methods:**

Polyethylene tubes filled with composite cement containing 10-methacryloyloxydecyl dihydrogen phosphate (10-MDP) were adhesively luted to 60 fully sintered Y-TZP slabs (7 x 7 x 2 mm) with or without (control) previous application of a 10-MDP-based adhesive (All Bond Universal, Bisco) – ABU; Clearfil Universal Bond Quick, Kuraray Noritake – CUB; Scotchbond Universal Adhesive, 3M Oral Care – SUA) on the zirconia surface. The bonded specimens were stored in water for 24 h, 6 months, or 1 year and subjected to microshear bond strength testing. The data were analyzed by one-way ANOVA and Tukey’s test (p < 0.05). The contact angle was measured after adhesive application to evaluate surface wettability. The adhesive-treated specimens were analyzed with x-ray photoelectron spectroscopy (XPS) and time-of-flight secondary ion mass spectrometry (ToF-SIMS) for chemical characterization.

**Results:**

The application of a 10-MDP-based adhesive significantly improved bond strength of composite cement to zirconia when compared to the control group (no adhesive application) (p < 0.05). One-year water storage significantly decreased bond strength for ABU- and CUB-bonded specimens, but not for SUA-bonded specimens. The analysis by XPS and ToF-SIMS showed peaks of carbon, phosphorus, and silicon in all adhesive-treated specimens.

**Conclusions:**

One-year water storage affected the bond strength of composite cement to zirconia when All Bond Universal or Clearfil Universal Bond Quick were used.

Yttria-stabilized tetragonal zirconia polycrystal (Y-TZP) is a ceramic widely used for dental applications due to its excellent physical and mechanical properties.^13,25^ However, bonding to Y-TZP remains a challenge, because its high crystallinity and the absence of a glass phase makes the use of conventional treatments recommended for glass ceramics, such as hydrofluoric acid and silane, ineffective.^45,54,56^ Thus, alternative surface treatment methods to either increase surface roughness or promote chemical bonding have been investigated in an attempt to improve the bonding of Y-TZP restorations to the tooth structure.^8,28,45,47,56^

Alumina particle abrasion, grinding with diamond rotary instruments, selective etching infiltration, tribochemical silica coating, and Er:YAG laser irradiation are amongst the methods proposed to increase Y-TZP surface roughness.^12,29,40,43,56^ Although there is no consensus in the literature about the most successful surface treatment for zirconia, alumina-particle abrasion has been the most frequently used method to promote micromechanical interlocking between composite cement and zirconia.^12,13,29,60,61^ A clinical study evaluating the longevity of alumina-abraded anterior zirconia prostheses bonded with composite cement showed that only 6 out of 180 restorations debonded within 10 years.^33^ However, low in-vitro bond strengths are still reported for zirconia. Thus, it is still necessary to keep investigating the factors that play a role in the stability of the zirconia-composite cement interface.

The chemical interaction between Y-TZP and the composite cement is promoted through a phosphate-based coupling agent,^1,30,56^ since zirconia is a non-polar and inert material.^53^ A phosphate-based monomer commonly used as a coupling agent is 10-methacryloyloxydecyl dihydrogen phosphate (10-MDP), because its phosphate ester groups bond chemically to metal oxides,^2,18,42^ while the 10-MDP vinyl group copolymerizes with the composite cement.^34,41^ Manufacturers have incorporated 10-MDP into adhesives, primers, and composite cements for dental applications in an attempt to improve the bonding of composite cements to zirconia.^18,22,26,36,60,65^

Universal adhesives containing 10-MDP and silane were then developed to be used with multiple indirect restorative materials, such as zirconia, alumina, glass ceramics, and metals.^3,17,55^ Studies have shown that universal adhesives are as effective as or even more effective than primers for zirconia.^5,18,22,34,50^ The versatility of universal adhesives, reducing clinical steps and cost, accounts for the good acceptance on the dental market.^3^ However, universal adhesives contain hydrophobic and hydrophilic components that may compromise the effectiveness and durability of the bond to zirconia.^11,23,35,37,64^ For this reason, studies are needed to assess the effectiveness of these adhesives over time.

The objectives of this in-vitro study were to evaluate: the effect of universal adhesives on bond strength of an 10-MDP-containing composite cement to zirconia; the wettability of the zirconia surface by different universal adhesives; and the effect of storage time on bond strength of universal adhesives to zirconia. Adhesive-treated specimens were also analyzed by x-ray photoelectron spectroscopy (XPS) and time-of-flight secondary ion mass spectrometry (ToF-SIMS) to investigate the chemical characteristics of the bonded interface to zirconia. The null hypotheses tested were: (1) bond strength is not affected by the application of universal adhesives; (2) storage time has no effect on bond strength of composite cement to zirconia, regardless of the adhesive used; (3) universal adhesive application does not affect the wettability of the zirconia surface.

## MATERIALS AND METHODS

### Specimen Preparation

**Fig 1:**
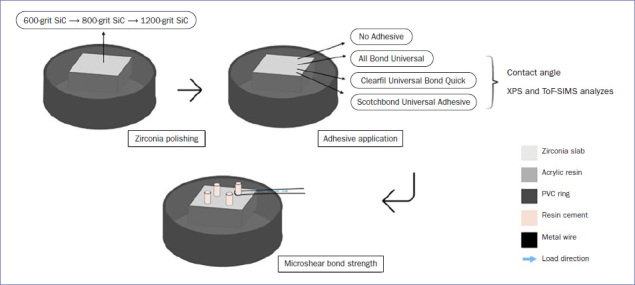
Schematic illustration of specimen preparation and experimental design.

3Y-TZP blocks (IPS e.max ZirCAD inLab MO C13, Ivoclar Vivadent; Schaan, Liechtenstein) were cut with a diamond blade (Series 15LC Diamond, Isomet, Buehler; Lake Bluff, IL, USA) at 500 rpm under water irrigation, to generate 60 square slabs. After being fully sintered according to manufacturer’s instructions, the slabs (7 x 7 x 2 mm) were embedded in auto-polymerizing acrylic resin (Jet Clássico; São Paulo, Brazil). The slabs were polished with silicon-carbide paper (grit sizes #600, #800, and #1200) under water using a rotary polisher (DP-10, Panambra; São Paulo, Brazil) to standardize the bonding surface of zirconia specimens (Fig 1). Subsequently, the slabs were cleaned in an ultrasonic bath with 99% ethanol for 5 min and dried with oil-free air spray for 15 s. All sixty zirconia slabs were randomly assigned to 4 groups (n = 15) according to the universal adhesive applied as surface treatment: CT (control) – no adhesive; ABU – All Bond Universal; CUB – Clearfil Universal Bond Quick; SUA – Scotchbond Universal Adhesive (Fig 1).

To prepare microshear test specimens, each zirconia slab received adhesive treatment as per corresponding experimental group. Details of the material composition and application technique for ABU, CUB, and SUA are presented in Table 1. The adhesives were light cured for 10 s with a light emitting diode (LED) device (Radii-Cal; SDI Dental Products; Bayswater, Victoria, Australia) operating at an irradiance of 1200 mW/cm^2^.

**Table 1:** Composition and instructions for application of the adhesives used in the different experimental groups

Code Brand name (manufacturer), lot number	Composition	Manufacturer’s instructions
ABU All Bond Universal (Bisco; Schaumburg, IL, USA), 1600358372	10-MDP, bis-GMA, HEMA, ethanol, water, initiators	Apply 1 coat of All-Bond Universal and air dry to remove excess solvent. Light cure for 10 s.
CUB Clearfil Universal Bond Quick (Kuraray Noritake; Tokyo, Japan), 2F0022	10-MDP, bis-GMA, HEMA, hydrophilic amide monomers, colloidal silica, silane sodium fluoride, dl-camphorquinone, ethanol, water	Apply Clearfil Universal Bond Quick to the adherent surface. Dry the bond with mild air blowing for more than 5 s until the mixture does not move; light cure.
SUA Scotchbond Universal Adhesive (3M Oral Care; St Paul, MN, USA), 80209B	10-MDP, dimethacrylate resins, HEMA, functionalized methacrylate, polyalkenoic acid (Vitrebond Copolymer), filler ethanol, water, initiators, silane	Apply Scotchbond Universal Adhesive to the restoration for 20 s. Air dry for 5 s and light cure.

HEMA: 2-hydroxyethyl-methacrylate; bis-GMA: bisphenol A glycidyl methacrylate; 10-MDP: 10-methacryloyloxydecyl dihydrogen phosphate.

Four polyethylene tubes with a diameter of 0.8 mm and a height of 1 mm (Tygon tubes, Odeme Biotech; Joaçaba, SC, Brazil) were placed on the surface of each zirconia slab with the aid of perforated double-stick tape and used as matrices for the composite cement. Dual-cure composite cement (Panavia F 2.0, Kuraray Noritake; Osaka, Japan) was mixed according to the manufacturer’s instructions and carefully injected into each matrix with a syringe. A mylar strip was placed over each filled tube and pressed gently. The composite cement was light activated for 20 s.

After 24 h of storage in distilled water at room temperature, the matrices were carefully removed with #11 scalpel blades to expose the composite cement cylinders. Each specimen was examined in a stereomicroscope (HMV-2, Shimadzu; Tokyo, Japan) at 40X magnification, and if any defect was observed, the specimen was discarded. Sixty composite cement cylinders were obtained for each group. Specimens were aged by storing in distilled water at room temperature (~22ºC) different durations (see below). The water was replaced weekly.

### Microshear Bond Strength Test (μSBS)

Three subgroups from each surface treatment, each with 20 composite cement cylinders, were stored in water for 24 h, 6 months, or 1 year. The power calculation of sample size showed that 12 specimens per group would be sufficient to ensure power adequate for detecting statistical significance (0.80).

The composite cement/zirconia interface was subjected to shear stresses with a thin metal wire (0.2 mm diameter) looped around the composite cement cylinder (Fig 1). Tensile load was applied in a universal testing machine (Instron 4444; Canton, MA, USA) at a crosshead speed of 0.5 mm/min until failure occurred. The μSBS (MPa) was calculated by dividing the load at failure by the surface area (mm^2^) of each specimen.

All specimens were examined under a stereomicroscope at 40X magnification to identify failure mode. The failure mode was classified according to the composite cement remaining on the zirconia surface: <1/3: adhesive failure; >1/3 but <2/3: mixed failure; > 2/3: cohesive failure.^39^

### Contact Angle

Using a goniometer (Easy Drop Contact Angle-Kruss; Hamburg, Germany), the contact angles of universal adhesives (ABU, CUB, SUA) to Y-TZP surfaces were analyzed to determine the wettability of the zirconia surface by each material using the sessile drop technique. A drop of each universal adhesive was applied on a clean zirconia surface and water was used as the control treatment. For each group, three measurements were performed on each zirconia slab (n = 5).

### X-ray Photoelectron Spectroscopy (XPS)

Additionally, one zirconia slab (7 x 7 x 2 mm) per group was prepared for chemical analysis of each adhesive using XPS. After surface finishing, cleaning, and drying the specimens as previously described, each universal adhesive was applied to the surface of one specimen and light cured as instructed by the manufacturers. Specimens were placed into the chamber of the XPS spectrometer (PHI 5000 Versaprobe III Scanning X-Ray Photoelectron Spectrometer, Ulvac Phi; Kanagawa, Japan) under ultra-high vaccum (<10^-8^ torr) and the settings were adjusted based on a 2 x 2 mm field of view area on the specimen’s surface. The spectrophotometer was equipped with a 180° hemispherical electron energy analyzer and a monochromatized Al Kα (1486.6 eV) source operated at 4 kV under a current of 4 µA. The depth profile was analyzed with an Ar+ ion gun at a sputtering rate of 28.5 nm/min. The maximum depth achieved in all specimens was 4 µm. Surveys were performed before and after the depth profile analysis.^63^ The focused x-ray beam had an angulation of 44.7° in the chamber with the internal platform. Consequently, the surface of the specimens received the beam at an angle in order to reflect the electrons into another chamber for the electron energy analyzer (binding energy – eV).

### Time-of-Flight Secondary Ion Mass Spectrometry (ToF-SIMS)

After depth profiling using XPS, zirconia specimens were examined with ToF-SIMS (PHI TRIFT V nano TOF, Physical Electronics, Ulvac-Phi). The craters created at the center of specimens by the XPS analysis were kept intact, allowing scanning in the ToF-SIMS to identify any additional chemical compounds. No additional sputters were made on the surface of the specimens for ToF-SIMS depth profiling. Equipped with a 20-Kv C60 ion gun for organic compounds analysis, the spectrometer was set with a beam energy of 25 KeV, DC current of 1.6 nA, frequency of 8200 Hz, and pulse width of 16 ns. The ion beam scanned the center of the crater to obtain 30 x 30 µm^2^ mass data in a sputter time of 10 min for each specimen. A negative ion, ZrO_2_^−^(121.9), was identified as the characteristic ion of the zirconia substrate. The ion of trimethoxysilyl group SiO_3_C_3_H_9_^−^(121) was the characteristic peak of silane, while 10-MDP was identified by PO_2_^−^(63) and PO_3_^−^(79) peaks.^15^

### Statistical Analysis

To test the normality of data distribution, Shapiro-Wilk (p = 0.13) and Levene’s homogeneity tests (p = 0.68) were employed. Microshear bond strength data were analyzed using one-way ANOVA and Tukey’s post-hoc test (p < 0.05). Contact angle results were also analyzed using one-way ANOVA and Tukey’s post-hoc test. For the test, a significance level of 5% was pre-set.

## RESULTS

### Microshear Bond Strength

One-way ANOVA indicated a significant effect of adhesive (p = 0.004) and storage time (p < 0.0001) on the bond strength between composite cement and zirconia. Means, standard deviations, and Tukey’s post-hoc test results for μSBS are presented in Table 2.

**Table 2:** μSBS bond strengths in MPa (mean ± SD) and results of Tukey’s post-hoc test

Groups	24 h	6 months	1 year
CT	8.9 (4.1)^A^	_*	_*
ABU	14.1 (4.7)^Ba^	10 (3.0)^Aab^	6.7 (4.3)^Ab^
CUB	17.6 (4.9)^Ba^	9.2 (3.9)^Ab^	8.7 (3.3)^ABb^
SUA	15.3 (4.0)^Ba^	10.3 (4.3)^Ab^	12.1 (4.4)^Bab^

*All specimens debonded spontaneously during water storage. Different uppercase letters within each column and lowercase letters within each row indicate significant differences between experimental conditions (p < 0.05).

**Fig 2:**
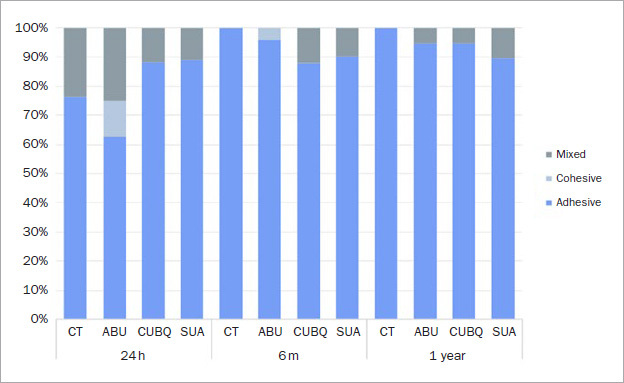
Mode of failure for each group (%) after 24 h, 6 months, and 1 year of storage in water.

Control specimens without adhesive application spontaneously debonded prior to testing after 6-month storage; thus no control specimen survived 1 year of storage. Water storage significantly affected bond strength of both ABU and CUB to zirconia after 1 year. Bond strengths of SUA significantly decreased after 6 month, but there was no difference between initial bond strength and after 1-year storage. Failure mode distribution is presented in Fig 2. The predominant mode of failure for all treatments and storage times was adhesive.

### Contact Angle

Contact angles for the three universal adhesives and water (control) are shown in Table 3. The control group showed significantly greater contact angles than those of the universal adhesives (p < 0.001). ABU and SUA resulted in similar contact angles, which were lower than those presented by CUB.

**Table 3:** Contact angle results

Group	Contact angle (mean ± SD)
Control	71.5 (2.0)^A^
ABU	31.2 (0.5)^C^
CUB	44.7 (1.7)^B^
SUA	34.5 (3.4)^C^

Different letters indicate significant differences between experimental groups (p < 0.001).

### X-ray Photoelectron Spectroscopy (XPS)

**Fig 3:**
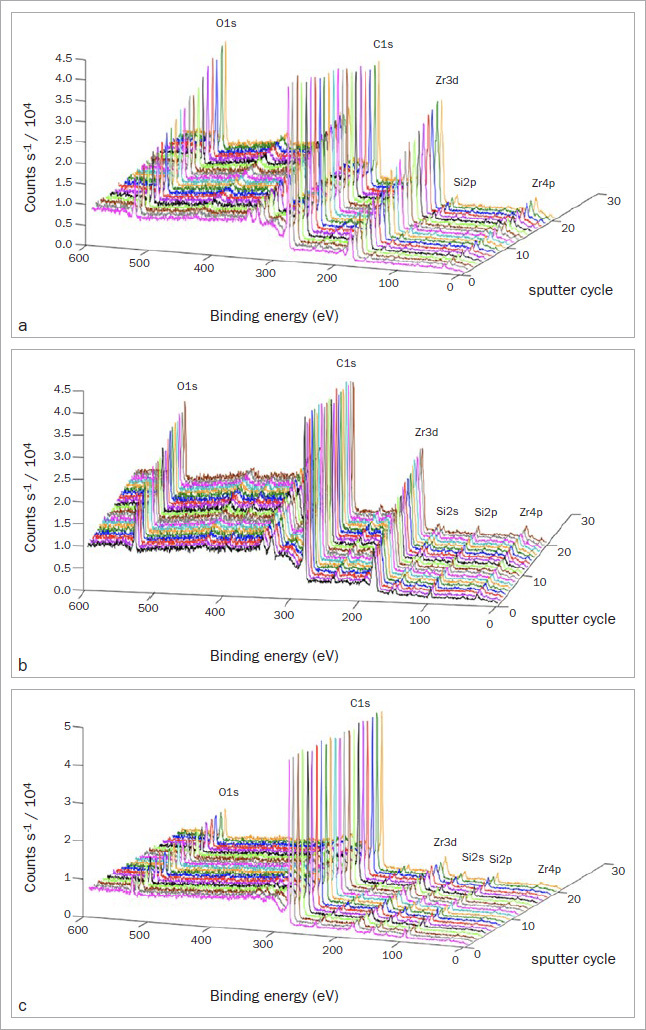
XPS results of the adhesive-treated specimens, indicating different spectral levels of O1s, C1s, Zr3d, Si2p, Si2s and Zr4p along a depth profile of 4 µm. a: ABU; b: CUB; c: SUA.

XPS revealed that the C1s carbon spectrum represents both HEMA and bis-GMA, which are present in all the materials applied to the zirconia specimens. The chemical state initially identified was C-C (eV 284.8), and then a shift to C-O-C (eV ~286) and carbonyl groups was observed (O-C=O; eV ~288.5). The presence of silicon at the initial survey, identified by the presence of Si2p and Si2s, was stronger on SUA. CUB and ABU presented Si2p and Si2s when the depth profile analysis was conducted. P2p and P2s (phosphorus) peaks, which are linked to the phosphate bonds of the 10-MDP monomer present in the adhesives, indicated the presence of adhesive components tested on the zirconia surface. At the initial survey, the presence of P2p was stronger on CUB and remained stable as the depth profile was conducted. P2p was not as strong for ABU in the initial survey when compared to CUB, and it remained low throughout the depth profile when compared to the other two adhesives. The presence of Zr3d is related to the zirconia surface, and as the depth profile advanced in depth, the concentration of Zr3d increased across all materials. Nonetheless, it was possible to observe that SUA presented the thickest layer over the zirconia surface due to the presence of C1 in comparison with the presence of other elements (Fig 3).

### Time-of-Flight Secondary Ion Mass Spectrometry (ToF-SIMS)

**Fig 4:**
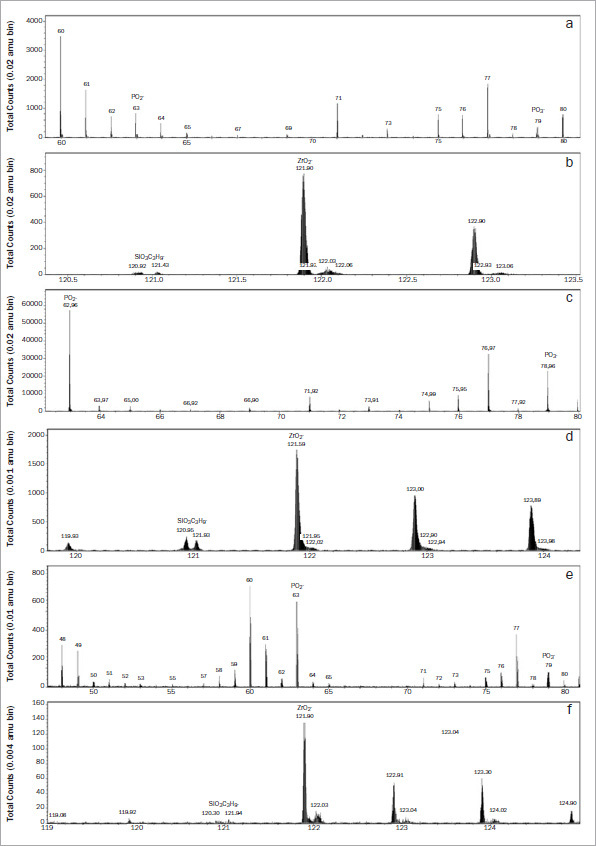
Negative secondary ions mass ToF-SIMS spectra of the adhesive-treated specimens. Peaks of ZrO_2_^–^(120 -125), SiO_3_C_3_H_9_^–^(121–122), PO_2_^–^(63) and PO_3_^–^(79) were observed in all evaluated 10-MDP-containing adhesives. a and b: ABU; c and d: CUB. e and f: SUA.

The analysis of the negative ion spectra for all the adhesives evaluated revealed the presence of the characteristic zirconia ion ZrO_2_^−^(121.9) from m/z 120 to 125. Additionally, the presence of SiO_3_C_3_H_9_^−^(121–122) indicates trimethoxysilyl (silane) on the surface of zirconia from all three universal adhesives. However, SUA only presented some traces of the trimethoxysilyl on the surface. The three adhesives showed various PO_2_^−^(63) and PO_3_^−^(79) distributions, and the peak intensity differed according to the surface treatment. CUB showed the greatest amount of PO_2_^−^(63) and PO_3_^−^(79) (Fig 4).

## DISCUSSION

To evaluate the bond strength between two different substrates, tensile or shear bond strength tests are commonly used both on the macro- and microscale.^6,7,10^ Conventional shear bond testing is usually used in dentistry to evaluate the bond strength, however the heterogeneity of the stress distribution and the incidence of cohesive failures have undermined the validity of the test.^10,21,58,59^ Sano et al^48^ observed that the area of the bonded interface is inversely correlated with the bond strengths. Therefore, microscale tests were introduced to evaluate the bond strength of adhesive interfaces in specimens with reduced dimensions, aiming to generate more reliable values.^27,48^ In general, the microshear bond strength test has been preferred by researchers due to its simplicity and low cost in comparison to the microtensile test.^6,7,24^

In the present study, the microshear bond strength test was performed to evaluate the effect of universal adhesives on the initial and long-term bond strength between a 10-MDP-containing composite cement and zirconia. Although alumina-particle abrasion is considered a standard method to improve the bond strength of composite cements to zirconia,^9,60,62^ no mechanical surface treatment was performed, aiming to isolate the effect of the chemical treatment on the bond strength, as reported in other studies.^18,23,34,36^

Some studies have suggested that 10-MDP-containing primers and adhesives are essential to improve the bond strength of composite cement to zirconia,^2,18,19,57^ while others observed that the bond strength to zirconia is not affected by the presence of 10-MDP within the adhesive.^26,51^ In the present study, when a 10-MDP-containing composite cement was applied without previous application of an 10-MDP-containing adhesive, significantly lower initial bond strengths were observed (Table 2). This leads to the rejection of the first null hypothesis. These findings may be related to different factors. First, contact angle results indicated the poor wettability of zirconia by water under control conditions (Table 3).^38^ Zirconia’s low surface energy coupled with the high viscosity of the composite cement when compared to the universal adhesives probably impaired the development of an effectively adhesive interface between zirconia and composite cement.^20^ Second, the lower availability of 10-MDP when only a composite cement was employed may also have limited the development of chemical bonds to the zirconia structure.

Storage in water and thermocycling are the most common methods used in the literature to simulate the aging of the bonded interface^18,35,37,44,46,65^ and they seem to effectively simulate aging in-vitro.^42^ Therefore, all specimens were subjected to storage in water for up to 1 year. Long-term water storage is important to better predict the stability of resin-based materials in the oral environment over time.^4^

One-way ANOVA showed an effect of storage time on bond strength of composite cement to zirconia. Therefore, the second null hypothesis was partially accepted. In this study, the bond strength of the control group specimens (no application of a universal adhesive) was not stable over time, with all specimens debonding spontaneously before 6 months of water storage, similar to the results of other studies.^32,60,65^ Water sorption and hydrolytic degradation of the adhesive interface seem to be the main reasons for this failure,^3,4,14,49^ although the composite cement employed contained 10-MDP. The permeability of the composite cement/zirconia interface induces the hydrolysis of the bond between 10-MDP and zirconia, which is responsible for the degradation of the bonded interface.^14^

The analysis of results indicated that storage in water also significantly affected the bond strength of adhesives/composite cement to zirconia after 1 year, except for SUA. Although the bond strength of ABU and CUB groups decreased after 1-year storage in water, values were still higher than 5 MPa, which is the minimum threshold for bond strength defined by ISO 10477.^31^ The 4-µm depth profile used with XPS indicated that all universal adhesives used in this study had phosphorus (P2s and PSp) peaks, which are indicators of the presence of 10-MDP in the material.^18,37^ XPS analysis also detected bis-GMA and HEMA in all the adhesives evaluated, reflected in the presence of C1s carbon spectrum. HEMA is frequently added to adhesives because of its solvent nature, although researchers claim that HEMA is hydrolytically unstable.^3,52^ HEMA is a hydrophilic monomer with a high water sorption capacity, which may have contributed to the hydrolytic degradation of the adhesive interface, thus reducing the bond strength over time.^3,11^ Nonetheless, the bond strength of SUA to zirconia was not affected by 1-year water storage. SUA contains a unique component (Vitrebond) that is not present in the other adhesives (ABU and CUB). A previous study reported higher bond strength of SUA/composite cement to zirconia after aging, and mentioned that the presence of polyalkenoic acid (Vitrebond copolymer) may be responsible for the higher hydrolytic stability of the adhesive.^65^ Another factor that may have reduced the effect of aging at the SUA/zirconia interface was the higher wettability of zirconia by this adhesive (Table 3), which resulted in significantly lower contact angles than the control and CUB groups. This wettability may have delayed the hydrolytic degradation due to the effective chemical interaction between the two substrates – adhesive and zirconia.

The chemical bond between 10-MDP-containing adhesives and zirconia was analyzed using ToF-SIMS. All adhesives tested showed peaks of PO_3_^−^, which is the elemental ion involved in the formation of P–O–Zr bonds.^15^ The highest amount of PO_3_^−^ was observed for CUB. The results of the XPS analysis also revealed a higher concentration of phosphorus in the CUB-treated surfaces, indicating a greater concentration of 10-MDP. Although more P–O–Zr bonds may have formed with CUB, the initial bond strengths did not different from those of ABU and SUA groups. ABU and SUA presented lower phosphorus and PO_3_^−^ peaks according to XPS and ToF-SIMS analysis. The similar initial bond strengths may be a consequence of variations in the surface wettability. Although CUB presented a higher 10-MDP concentration, it resulted in the highest contact angle to zirconia (Table 3), which may have counteracted any potential benefit of the higher 10-MDP concentration. Thus, the third null hypothesis was also rejected. Although Nagaoka et al^41^ observed that the bond strength to zirconia increased with higher 10-MDP concentration up to 1 wt%, Lorena et al^36^ showed that the presence of 10-MDP in the adhesive for bonding to zirconia was more influential than the concentration (3 wt%-15% wt%). In these previous studies, no long-term storage or aging of any kind was performed, but it has been reported that bonding to zirconia is negatively affected by aging.^16,23,34^

XPS and ToF-SIMS results showed peaks of Si2p/Si2s and SiO_3_C_3_H_9_^–^(121); these were the components associated with the presence of silane. Si2p/Si2s and SiO_3_C_3_H*9*^–^(121) were present in all adhesives evaluated in this study. Although silane has no chemical affinity to the untreated surface of zirconia, it is present in universal adhesives to mediate chemical interactions between other materials and substrates. Previous studies reported that silane may increase the hydrophilicity of adhesives and contribute to the hydrolytic degradation of the adhesive layer.^23,34,55^ Interestingly, SUA-treated specimens showed the lowest peak of SiO_3_C_3_H*9*^–^(121) and the highest bond strength after 1 year of water storage, indicating a potential stability of the adhesive due to the low amount of silane.

The stability of the bond of composite cement to zirconia is an important factor to ensure the longevity of zirconia restorations.^56^ Within the limitations of this in-vitro study, one year of water storage may be considered a long-term test of bond stability. However, simple storage in water is not able to replicate the aging of the bonded interface with the fidelity required to estimate long-term intraoral bond strength. The oral cavity is an environment presenting various challenges, such as constant changes in pH, temperature, and chewing loads, that were not reproduced in this work, but should be considered in future studies.

## CONCLUSIONS

One-year water storage negatively affected the bond strength of composite cement to zirconia when All Bond Universal or Clearfil Universal Bond Quick were used.

## Clinical relevance:

Surface treatment with an MDP-based adhesive improves the bond strength of an MDP-containing composite cement to zirconia. However, the bond strength is prone to decrease over time.
